# Intestinal Barrier Impairment, Preservation, and Repair: An Update

**DOI:** 10.3390/nu16203494

**Published:** 2024-10-15

**Authors:** Ayah Matar, John A. Damianos, Kara J. Jencks, Michael Camilleri

**Affiliations:** Clinical Enteric Neuroscience Translational and Epidemiological Research (CENTER), Division of Gastroenterology and Hepatology, Mayo Clinic, Rochester, MN 55905, USA; matar.ayah@mayo.edu (A.M.); damianos.john@mayo.edu (J.A.D.);

**Keywords:** permeability, fat, emulsifier, tight junctions, epigenomics, inflammation

## Abstract

Background/Objectives: Our objective was to review published studies of the intestinal barrier and permeability, the deleterious effects of dietary components (particularly fat), the impact of altered intestinal permeability in disease models and human diseases, the role of the microbiome and epigenomics in control of barrier function, and the opportunities to restore normal barrier function with dietary interventions and products of the microbiota. Methods: We conducted a literature review including the following keywords alone or in combination: intestinal barrier, permeability, microbiome, epigenomics, diet, irritable bowel syndrome, inflammatory bowel disease, probiotics. Results: Intestinal permeability is modified by a diet including fat, which increases permeability, and nutrients such as fiber, glutamine, zinc, vitamin D, polyphenols, emulsifiers, and anthocyanins, which decrease permeability. There is significant interaction of the microbiome and barrier function, including the inflammatory of luminal/bacterial antigens, and anti-inflammatory effects of commensals or probiotics and their products, including short-chain fatty acids. Epigenomic modification of barrier functions are best illustrated by effects on junction proteins or inflammation. Detailed documentation of the protective effects of diet, probiotics, prebiotics, and microbiota is provided. Conclusion: intestinal permeability is a critical factor in protection against gastrointestinal diseases and is impacted by nutrients that preserve or heal and repair the barrier and nurture anti-inflammatory effects.

## 1. Introduction

The objective of this review is to provide information regarding the intestinal barrier and permeability, the deleterious effects of dietary components (particularly fat), the impact of altered intestinal permeability in disease models or human diseases, the role of the microbiome and epigenomics in the control of barrier function, the opportunities to restore normal barrier function with dietary interventions, and products of microbiota.

### Intestinal Barrier and Permeability

The gastrointestinal tract features one of the longest barriers between the environment and the systemic circulation, spanning up to 40 square meters. This barrier is composed of a mucus layer, commensal microbiota, the intestinal epithelium, and immune cells situated in the lamina propria [1,2,3]. The mucus layer over the intestinal epithelium safeguards it from contents within the intestinal lumen [4]. Commensal microbiota support barrier function, shape intestinal and systemic immune responses, and inhibit pathogenic bacteria by directly stimulating epithelial cells, producing nutrients and metabolites that are essential for enterocyte health, and influencing the priming of the immune system [5]. The epithelial semi-permeable barrier contributes to maintaining intestinal homeostasis by selectively allowing nutrients to cross from the intestinal lumen into the internal milieu, to reach the systemic circulation, while controlling the passage of bacterial pathogens, allergens, and toxins [2].

The epithelial cells are organized into a single layer of cells from four primary lineages: enterocytes (the most abundant), goblet cells, enteroendocrine cells, and Paneth cells [4,6,7]. Enterocytes are chiefly involved in absorption of nutrients, water and electrolytes from the lumen, goblet cells secret mucus that adds protection to the barrier layer, enteroendocrine cells secrete hormones that have diverse regulatory functions, and Paneth cells release antimicrobial peptides [2,6,8]. These cells function synergistically to maintain the gut barrier’s integrity [6].

Tight junctions (TJs) are a series of transmembrane proteins that connect intestinal epithelial cells and are essential for maintaining barrier integrity, particularly when the intestines are distended [4]. The recent literature has reported three main proteins that make up TJs and contribute to their function. Proteins found in the cytoplasm beneath the TJ membrane include cingulin (CGN), guanine nucleotide exchange factors (GEFs), GTPase-activating proteins (GAPs), and zonula occludens proteins (ZOs) including ZO-1, ZO-2, and ZO-3 (which are also known as TJP1, TJP2, and TJP3, respectively) [9,10]. Other proteins that contribute to the permeability/barrier function of TJs comprise claudin polymers that are either barrier-forming or channel-forming. It is important to note that further interactions between claudin isoforms also exist but have yet to be established [9,11]. Additionally among the proteins that contribute to the permeability, barrier, and adhesion function of TJs, there is junctional adhesion molecule A (JAM-A, also known as F11R) [9].

These junctions are also crucial for regulating the transport of substances between the intestinal lumen and systemic circulation [12]; such transport is mediated in part by the permeability of TJs, leading to three types of functional gaps with different properties: pore pathway, leak pathway, and restricted pathway [13]. The pore pathway is of high capacity and charge-selective, permitting the passage of small ions and uncharged molecules (typically <8 Å); the leak pathway is low-capacity and nonselective (<100 Å); on the other hand, the unrestricted pathway is independent of the TJ and results from epithelial damage [9,12,14]. Damage to these pathways alters the barrier integrity and may contribute to the development of disease [15,16].

Intestinal permeability refers to the functional properties of the intestinal barrier and is characterized primarily by paracellular and transcellular transport mechanisms [7]. The paracellular route, regulated by tight junctions, allows the passage of ions, water, and large hydrophilic compounds [17], while the transcellular route facilitates the passage of proteins, sugars, amino acids, and bacteria [18], typically by carrier-mediated transport. Disruption in either route can affect barrier homeostasis, alter permeability and potentially lead to disease [3,18,19].

Various factors, including dietary components, genetic predisposition, medications (e.g., NSAIDs and antibiotics), alcohol consumption, strenuous physical activity, psychological or environmental stress, pregnancy, pathogens, systemic diseases, inflammatory conditions and cytokines, metabolic disorders including obesity, and surfactants (e.g., bile acids and emulsifiers) can alter barrier homeostasis [3,17,18,19,20] and increase permeability, leading to “leaky gut” [18,21]. This “leakiness” allows passage of microorganisms, allergens, and toxins inciting inflammation locally in the gut, and passage into the systemic circulation [3,21]. “Leaky gut” has been implicated in a wide range of systemic diseases (including type 2 diabetes mellitus, obesity), neuropsychiatric diseases (e.g., Alzheimer disease, Parkinson disease, autism spectrum disorders, major depressive disorder), and autoimmune disease (e.g., psoriasis, rheumatologic diseases, and uveitis), in addition to intestinal diseases (e.g., inflammatory bowel disease (IBD), irritable bowel syndrome (IBS), celiac disease) [1,4,18,20,22].

## 2. Epigenetic Mechanisms Related to Intestinal Permeability

Recent data suggest epigenetic mechanisms are involved in regulating intestinal permeability. Epigenetic mechanisms include how the environment alters the expression of DNA without changing DNA nucleotides. Examples of epigenetic mechanisms include DNA methylation, histone modification, non-coding RNA activity, and chromatin remodeling, which all affect how genes are expressed. Environmental exposures, stress, toxins, medications, development, aging, and diet can induce epigenetic changes. Environmental states with increased levels of stress are thought to effect change via epigenetic mechanisms, related to the corticotropin releasing factor/hypothalamic-pituitary-adrenal axis [23]. The published data on epigenetic changes on intestinal permeability are primarily from stressed animal models which have increased intestinal paracellular permeability and visceral hypersensitivity [23]. In a chronic preclinical stress model, which is relevant to pathophysiological states like irritable bowel syndrome, the mice had increased levels of cytokine IL-6, which is pro-inflammatory, and correlated to a decrease in tight junction proteins in the colon, documented by an inverse correlation of IL-6 level and the expression of occludins [24]. IL-6 acted via epigenetic mechanisms (as evidenced by increased methylation of the repressive histone H3K9) with an observed increase in paracellular permeability [24]. In biopsies taken from patients with diarrhea-predominant irritable bowel syndrome, there were increased levels of micro-RNA 29A and B and reduced levels of claudin-1 (CLDN1) and nuclear factor-kB-repressing factor (NKRF), which was replicated in a knockout animal model that documented the fact that increased micro-RNA 29A and B decreased the levels of CLDN1 and NKRF mRNA [25].

Network enrichment analysis of RNA-sequencing data in a chronic stress model of rats identified that super enhancers play an important role in intestinal barrier dysfunction [26]. Super enhancers are sites in the genome with increased areas for transcription factors to bind (enhancers) and enhance genetic expression of an associated gene up to one million base pairs away. Super enhancers have recently been considered an epigenetic mechanism as they regulate gene expression without modifying DNA. In particular, in the rat chronic stress model, it was shown that super enhancers played a role in the downregulation of adherens and intestinal epithelial tight junction genes (e-cadherin (CDH1), tight junction protein 3 or ZO-3 (TJP3), p-cadherin (CDH3), m-cadherin (CDH15), claudin-2 (CLDN2), claudin-3 (CLDN3), claudin-7 (CLDN7), and tight junction associated protein 1 or 4 (TJAP1)), which are linked to increased paracellular permeability [26]. The microRNAs involved in intestinal permeability include microRNA-16 (downregulated in IBS), microRNA 125b (downregulated in jejunal IBS-D tissue), microRNA 144 (upregulated in a colon IBS_D rat model), and microRNA-29a (upregulated in colon and duodenum of IBS and rat models) [23,27,28,29,30,31].

Irritable bowel syndrome is not the only phenotype where intestinal permeability and epigenetic mechanisms have been studied. Paracellular membrane permeability was influenced by DNA methyltransferase 3A activity (which is involved in DNA methylation) in the colon epithelial cells from patients with IBD in studies conducted in vitro [32]. Colon tissue samples and organoids from patients with ulcerative colitis had increased levels of IL1B mRNA and microRNA 200C-3p that decreased the expression of occludin and increased intestinal permeability [33]. In patients with systemic lupus erythematosus, there is also evidence of association of possibly increased intestinal permeability, with DNA methylation levels being highest in 926 CpG sites [34].

Additionally, the microbiome is also thought to contribute to local changes in intestinal permeability and maintenance of membrane barrier function [35,36,37]. Increased intestinal permeability can result from altered expression of tight junction related proteins with changes in intestinal microbiota profiles [38]. The host phenotype is influenced by the epigenome–microbiome axis. This includes how the environment and host genotype affect both the host epigenotype and the microbiome composition and activity. It is also known that the microbiome and epigenotype contribute to the host phenotype [39]. Microbiota can induce modification of gene expression through epigenetic mechanisms [39]. For example, disruption to the barrier function was repaired though the activation of epithelial histone deacetylase 3 (HDAC3) via microbiota-derived IP3 (inositol triphosphate) in a murine model and intestinal organoid model [37]. Histone deacetylases remove acetyl groups from histones (and non-histones), decreasing transcription. Short chain fatty acids (SCFAs) like butyrate, acetate, and propionate inhibit host histone deacetylases, leading to silencing of transcription, chromatin condensation, histone acylation, and DNA methylation [39].

Chronic stress and the microbiome are not the only ways via which epigenetic modulation affects intestinal permeability. Ingested substances (discussed more extensively in subsequent sections and Table 1 also influence epigenetic mechanisms. For example, anthocyanins, which are part of the flavonoid group of polyphenols, naturally occur in many colorful fruits and vegetables. Anthocyanins appear to play a role in epigenetic modulation of the expression of key membrane integrity proteins, such as ZO-1 and occludins, in animal models [40]. Zinc also influences the gene expression of tight junction proteins, claudin 1 and 2, through histone deacetylase activity and chromatin remodeling [41,42].

## 3. Effect of Food Substances and Disease States on Intestinal Permeability

### 3.1. Food Substances That Decrease Intestinal Barrier Integrity or Increase Permeability

In view of the most recent extensive literature regarding the deleterious effect of fats on intestinal barrier function, and the associations of Western diet on diverse diseases, Table 1 summarizes the effects of high-fat diet on intestinal barrier function [21,43,44,45,46]. The table shows that, in vivo, fat intake is associated with serological markers of increased permeability such as plasma lipopolysaccharide and serum endotoxin; in addition, there was a trend to increased permeability measured using the ratio of the urinary excretion of oral sugar probe molecules (lactulose and mannitol). There is also evidence, based on in vitro studies of modulators from human Peyer’s patches, that the emulsifier polysorbate-80 increased translocation of *E. coli*.

### 3.2. Altered Permeability and Disease States

Altered permeability has been investigated in vivo in preclinical models of disease, as well as in human disease states, and these are summarized in Table 2 and Table 3 and elaborated further in Section 4 [21,47,48,49,50,51,52,53,54,55].

## 4. Effects of Dietary Components That Enhance or Damage the Intestinal Barrier

### 4.1. Nutrients

Table 4 provide a summary of the nutrients that either increase or decrease intestinal permeability as well as the mechanisms proposed for the changes in intestinal permeability

#### 4.1.1. Substances That Help Increase Intestinal Barrier Integrity

Numerous strategies have been suggested to address gut barrier dysfunction, such as direct immune therapies, blocking signaling factors, introducing specific microbes, fecal microbiota transplantation, using microbial metabolites, probiotics, targeting disease-induced regulatory factors, hormone treatments, treating underlying conditions, dietary supplementation, and maintaining a healthy diet and overall wellness [71]. This section focuses on dietary factors and microbial metabolites and nutrients, based on a review of the recent literature and updated versions of reports written by Khoshbin and Camilleri [42] and Camilleri and Vella [21].

#### 4.1.2. Dietary Fiber

Dietary fiber is a carbohydrate polymer made up of 10 or more monomers that are neither absorbed nor digested in the small intestine [56,57]. The four subgroups of dietary fiber that research primarily focuses on are resistant oligosaccharides, non-starch polysaccharides, resistant starches, and associated substances [57]. Dietary sources of resistant oligosaccharides include various legumes, vegetables, fruits, and specific plant products like chicory root and soybeans. Non-starch polysaccharides, such as cellulose, hemicellulose, and pectins are found in cereals, grains, fruits, and vegetables, while resistant starches come from foods like grains, green bananas, and processed products. Additionally, associated non-carbohydrate substances like lignin, waxes, and chitins are present in cereal grain outer layers, insect secretions, and the exoskeletons of crustaceans. These types of fiber are considered as prebiotics since stimulate bacteria beneficial for the gut health and aid in the increase of short chains of fatty acids (SCFAs) which also enhances the integrity of the intestinal barrier [47].

SCFAs, produced from dietary fiber fermentation by gut microbiota, play a crucial role in colon health and microbiota–gut–brain communication, providing 5–10% of human basal energy needs [58,59]. Research has focused around three SCFAs: acetate, butyrate, and propionate [60]. Acetate, the most abundant SCFA, supports ATP production, while SCFAs overall lower colon pH to inhibit pathogenic bacteria and modulate inflammation through various cellular mechanisms [60,61]. Butyrate, in particular, has significant health benefits, including cancer prevention, while propionate may reduce blood cholesterol, and both contribute to communication along the microbiota–gut–brain axis due to their neuro-active properties [62].

Resistant oligosaccharides include fructo-oligosaccharides (short chain inulin), which are β-(2 → 1) linear fructans derived from enzymatic hydrolysis of long-chain inulin or synthesized from sucrose [58]. Galacto-oligosaccharides consist of two to eight saccharide units with glucose and galactose and can be found in human milk or synthesized from lactose [58]. Other resistant oligosaccharides include non-digestible types like konjac oligosaccharide. Incorporating a wide range of fruits, vegetables, various legumes, and soybeans (resistant oligosaccharide products) can go along way toward enhancing gut health, especially considering recent Westernized diets that have become widely popular. Mistry et al. showed in an in vivo study on male mice fed a Western-type diet that resistant oligosaccharide supplementation significantly reduced bodyweight gain, fat accumulation, insulin resistance, and plasma cholesterol levels, while also altering gut microbiota composition in a way that may benefit metabolic health. These findings suggest that resistant oligosaccharides could have therapeutic potential for improving metabolic markers and reducing the risk of metabolic syndrome [47].

Non-starch polysaccharides include cellulose, the main structural component of plant cell walls, and hemicelluloses, which are diverse cell-wall polysaccharides found in fruits, vegetables, and cereals [58]. Pectins, found in plant cell walls, and various gums and mucilages from plants, microbes, and seaweed, are also key non-starch polysaccharides. Additionally, β-glucans and β-fructans, such as inulin from chicory roots, are non-digestible fibers that are resistant to enzymatic digestion but fermentable in the colon [58]. In an in vivo study with healthy male volunteers, inulin was shown to lower the lactulose–mannitol (L/M) ratio and serum zonulin while increasing mucosal GLP-2 after 8 weeks [21]. However, the study’s methods were suboptimal, including the use of inappropriate sugar probes, limited urine collection time, and serum levels that may not have accurately indicated intestinal permeability.

Resistant starches (RS) come in various forms, including physically inaccessible starch (RS1), granular native starch (RS2), retrograded starch (RS3), chemically modified starch (RS4), and resistant maltodextrins like Nutriose (RS5) [58]. Gondalia et al. [48] showed that in an in vivo study of 80 healthy adults who were either given high-amylose wheat (HAW) product or low-amylose wheat (LAW) product, HAW and LAW had similar effects on fecal output and total SCFA excretion, but the HAW-R group showed 38% higher fecal butyrate excretion and more SCFA-producing bacteria at 4 weeks. While LAW-R increased fecal p-cresol levels, which disrupts the epithelial barrier function, and also increased the abundance of a p-cresol-producing bacterium (*Clostridium difficile*, *Clostridium scatologenes*, *Clostridium bolteae*), these were reduced by HAW-R, with no impact on fecal consistency or digestive comfort from the amylose level [48].

#### 4.1.3. Polyphenols, Anthocyanins, and Ellagitannins

Recent studies have focused on polyphenols, particularly flavonoids (such as anthocyanins, flavonols, and flavanols), condensed and hydrolysable tannins (like ellagitannins), phenolic acids, stilbenes, and lignans, due to their potential benefits for the intestinal barrier [63,64,65]. These polyphenols are predominantly found in various types of berries, including blueberries, strawberries, and blackberries [65]. Berries are a rich source of flavonoids, particularly anthocyanins, which give them their vibrant colors and have strong antioxidant properties [65]. These pigments, along with flavonols and flavan-3-ols, are not only capable of crossing the blood–brain barrier but also offer potential health benefits, including cardioprotective, neuroprotective, anti-inflammatory, and anticancer effects [65].

Ellagitannins (ETs) are hydrolysable tannins abundantly found in raspberries, walnuts, strawberries, and pomegranate [64]. They are hydrolyzed in the intestinal lumen and release ellagic acid (EA), which is then further metabolized with ellagitannins into urolithins [64,66]. Due to the interindividual variability of metabolization of ETs, three Uro metabotypes (UM) that are microbiome-specific have been identified and are most relevant to this paper, metabotype A, metabotype B, and metabotype 0, yielding UM-A, UM-B, and UM-0, respectively [65,66].

Urolithin A (Uro-A) and to a lesser extent Urothilin B (Uro-B) exhibit anti-inflammatory, neuroprotective, cardioprotective, and anti-obesity activites, as well as improving gut microbiota and tight junction protein expression, cognitive function, and muscle function, while also demonstrating potential in reducing oxidative stress, protecting against organ damage, and modulating the immune response in various animal models [67]. A recent in vivo study found that administering Uro-A and Uro-B intraperitoneally for 4 weeks in rats on a high-fat diet altered gut microbiota composition, leading to a decrease in microbes associated with body weight, lipid metabolism issues, and inflammation [53].

#### 4.1.4. Glutamine

In an in vivo study investigating the effects of dietary glycyl-glutamine (GlyGln) supplementation, weaned piglets were given either a basal diet or the same diet supplemented with 0.25% GlyGln for 3 weeks [54]. Following intraperitoneal injections of lipopolysaccharide (LPS) to induce inflammation, the GlyGln-supplemented piglets showed improved ileum morphology, reduced inflammation, and enhanced oxidative status, with increased levels of interleukin 10 and tight junction proteins, as well as higher superoxide dismutase activity [54]. Additionally, GlyGln supplementation restored gut microbiota diversity and function, enriching beneficial bacteria and short-chain fatty acid producers, and improving intestinal integrity and microbial balance disrupted by the LPS challenge [54].

In another study that investigated the effects of oral supplementation with Ala-Gln and glutamine (Gln) on dextran sulfate sodium (DSS)-induced colitis in mice [55], both supplements significantly improved colitis symptoms, such as bodyweight loss and colon damage, reduced inflammation markers and tissue apoptosis, and enhanced gut microbiota diversity and function, with Ala-Gln demonstrating superior efficacy over Gln in preserving intestinal integrity and microbiota composition [55].

#### 4.1.5. Vitamin D

Research shows that vitamin D is essential for maintaining gut barrier integrity, with VDR-deficient mice study displaying increased susceptibility to bacteria and LPS in vivo due to weakened tight junctions [49]. Vitamin D supplementation strengthens the epithelial barrier by enhancing the expression of tight junction proteins like ZO-1, occludin, and claudin-1, as demonstrated in studies where 1,25(OH)2D3 treatment partially rescued these proteins’ reduced expression in DSS-treated Caco-2 cells [49,68]. Additionally, vitamin D upregulates antimicrobial peptides, which are crucial for maintaining a balanced microbiome and preventing harmful bacterial colonization [68]. Thomas et al. conducted an in vivo study where 567 men provided stool samples, to study their microbiome and correlate it with vitamin D metabolite levels. This study showed that men with the highest levels of active vitamin D (1,25(OH)2D) or activation ratios were more likely to have gut bacteria associated with butyrate production, which helps increase the integrity of the intestinal barrier, whereas serum 25(OH)D levels showed no strong association with microbiota diversity or specific bacteria [50]. This suggests that the regulation of vitamin D metabolism, rather than overall body stores of vitamin D, may have more significant health implications.

It is worth noting that Yamamoto et al. showed that improper regulation of vitamin D, whether through deficiency or excessive supplementation, can result in “leaky” junctions, which may partially contribute to the development of colitis [49]. They highlighted the importance of balancing vitamin D levels to ensure a therapeutic effect and not a damaging one [49]. However, more evidence is needed to fully understand the extent of vitamin D’s role in this process.

#### 4.1.6. Zinc

Recent studies have shown that zinc is a double-edged sword. In other words, both the deficiency and the overdosing of zinc can cause dysbiosis in the gut microbiome [69]. The deficiency of zinc has been correlated with multiple gastrointestinal disorders such as gastric cancer, Crohn’s disease, impaired intestinal permeability, and irritable bowel syndrome [51,70]. Omry et al. show in an in vivo study with chicks divided into two treatment groups based on sex and body weight, “high Zn” and “low Zn,” that Zn deficiency led to significant changes in microbial taxonomy, reduced species richness and diversity, and substantial reductions in KEGG pathways for nutrient uptake and beneficial short-chain fatty acids, which can impair optimal host Zn availability [51].

Consequently, supplementation with the appropriate dose of Zn has been shown to increase gut bacteria biodiversity, improve intestinal barrier integrity, and thereby limit the number of bacteria that can pass through the barrier into the systemic circulation [52]. The overdosing of Zn has been hypothesized to be related to the presence of “free flowing” Zn, thus altering immunity and causing dysbiosis [52]. An in vivo study in mice of chronic toxic exposure to ZnSO4 for 7 weeks showed reduced body and organ weight and increased AST activity [52]. Thus, excessive Zn doses can induce oxidative stress, compromise barrier integrity, and increase intestinal permeability, leading to a higher risk of the progression of other diseases [52,70]. Therefore, the right dosage of Zn should be provided and monitored to ensure its beneficial outcomes.

#### 4.1.7. Emulsifiers

Emulsifiers, commonly known as surfactants, are widely used as food additives in the food industry and are highly prevalent in ultra-processed foods [42]. Previous research conducted primarily on mice in vitro indicated that emulsifiers increased intestinal permeability and were proinflammatory [72,73,74,75]. The study details were as follows:

Levine et al. [72] and Bancil et al. [74] conducted reviews of the existing literature, including both in vivo and in vitro studies, which showed that emulsifiers such as carboxymethylcellulose (CMS) and polysorbate 80 (P80) disrupt the intestinal mucosal layer leading to increased intestinal permeability or “leaky gut”.

Naimi et al. studied the effects of CMC and P80 on the gut microbiota using in vitro fermentation of healthy human fecal samples. This study showed that exposure to either CMC or P80 caused a reduction in microbial diversity, an increase in pro-inflammatory bacteria (*Proteobacteria*), a decrease in butyrate production, decreased barrier integrity, and increased permeability in an in vitro epithelial model.

Ogulur et al. [75] conducted both in vitro and in vivo studies with polysorbate 20 (P20) and P80. In vitro studies were carried out on human intestinal epithelial cell lines (Caco-2 and HT29 cells), indicating that P80 and P20 compromised barrier function and disrupted TJ proteins. In vivo studies were conducted on mice and also showed an increase in intestinal permeability.

However, a recent in vivo and in vitro study by Fitzpatrick et al. involving 22 healthy adults who were fed either a high- or low-emulsifier diet (CMC, P80) produced unexpected results [76]. The study found that, based on diverse measurements (specifically, 2 h urinary lactulose–rhamnose ratio, serum concentrations of lipopolysaccharide-binding protein, soluble CD14, markers of epithelial injury and inflammation), diets high in emulsifiers actually improved intestinal barrier function in unstressed conditions, but increased sensitivity of intestinal permeability in response to stress [76]. Neither effect was associated with signs of inflammation [76]. More studies should be conducted to further solidify this finding and to appraise the interaction of experimental stress and exposure to emulsifiers.

### 4.2. Microbial Nutrients and Metabolites

#### 4.2.1. Prebiotics

Descriptions and Mechanisms of Action: Prebiotics are defined as “substrate[s] that [are] selectively utilized by host microorganisms conferring a health benefit” [77]. While many different classes of compounds function as prebiotics, the most common types are poorly or non-digestible carbohydrates that are resistant to endogenous intestinal digestive enzymes. Non-digestible oligosaccharides represent a broader category that includes any oligosaccharides that are not digested nor absorbed in the small intestine; this group encompasses resistant or non-resistant oligosaccharides from the perspective of microbial fermentation. These oligosaccharides, such as fructans and galactans, reach the colon and are selectively metabolized by beneficial bacteria such as bifidobacteria. For instance, fructo-oligosaccharides (FOSs) and galacto-oligosaccharides (GOSs) are not digestible by human enzymes and can serve as substrates for fermentation by beneficial gut bacteria. Thus, they are degraded by β-fructanosidase and β-galactosidase enzymes, respectively. This selective utilization results in the production of short-chain fatty acids (SCFAs) such as acetate, propionate, and butyrate, which are crucial for maintaining intestinal health and can influence metabolic activities, immune functions, and overall homeostasis. Other examples of prebiotics include human milk oligosaccharides (HMOs), which are vital for the development of the newborn intestinal microbiota and immune system. HMOs are selectively utilized by specific *Bifidobacterium* species, promoting the growth of beneficial bacteria and protecting against pathogens. Plant polyphenols are another class of compounds that meet prebiotic criteria, undergoing extensive biotransformation in the colon to produce health-promoting metabolites [77].

Prebiotics exert significant protective effects on the intestinal barrier through multiple mechanisms. One primary effect is the modulation of microbial communities within the gut. Fermentation of prebiotic fibers leads to the production of SCFAs, which play a crucial role in maintaining the integrity of the intestinal barrier. Butyrate, in particular, is a key energy source for colonocytes and helps strengthen the epithelial barrier by promoting mucus production and secretion. This mucus layer acts as a protective shield, preventing microbial invasion and reducing susceptibility to infections and inflammatory conditions. Additionally, acetate and propionate support goblet cell function and mucin production, further enhancing the barrier function [78]. Prebiotics can also enhance the intestinal microenvironment in mechanisms independent of SCFAs. For instance, fiber can bind and transport essential minerals like calcium, zinc, and copper to the distal gut, where they are released for absorption. This process not only supports the microbiota but also improves mineral bioavailability for the host. Moreover, fiber may facilitate interactions between bacteria and biomolecules such as bile acids, enhancing microbial metabolism [78]. In addition to a large body of pre-clinical evidence detailing the mechanisms of prebiotics, there is also considerable pre-clinical evidence that these effects are anti-inflammatory in the intestines. For example, numerous studies have found that prebiotics can reduce inflammation in animal models of colitis [79]. A comprehensive summary of clinical trials that have assessed the effects of prebiotics on intestinal permeability is presented in Table 5 [3,80,81,82,83,84,85,86,87,88,89,90,91,92].

In summary, the evidence for prebiotics to reduce intestinal permeability is mixed. Some studies, such as those involving formula-fed infants and healthy young men, demonstrated significant reductions in intestinal permeability with prebiotic supplementation. However, other studies, particularly those involving vulnerable populations like preterm infants, burn patients, and individuals with type 2 diabetes or obesity, showed no significant improvements in intestinal permeability. The methodologies for assessing intestinal permeability varied across studies, with most involving the lactulose–mannitol ratio or other sugar-based tests. Other heterogeneity among the studies resulted from study design, population variability, and differences in prebiotic formulations (as well as dosing and duration). While prebiotics show promise in reducing intestinal permeability, further research is needed to identify which populations benefit most and the optimal prebiotic formulation for the given indication.

#### 4.2.2. Probiotics

Descriptions and Mechanisms of Action: Probiotics are defined as “live microorganisms which when administered in adequate amounts confer a health benefit on the host” [93]. They encompass a wide range of single and multi-strain formulations of microorganisms (usually bacteria and/or fungi). While many of the specific health benefits derived from probiotics are strain-specific, there are general mechanisms thought to be shared among the most common probiotic organisms. These include the production of active metabolites (such as short-chain fatty acids, SCFAs), secretion of compounds (such as secretory IgA), interaction with the immune system, contribution to digestion and bile acid metabolism, manufacturing of systemically active molecules (such as serotonin), communication with the endogenous gut microbiota, and maintenance of the gut epithelial barrier [94]. Probiotics influence intestinal barrier function by modulating tight junction (TJ) proteins in intestinal epithelial cells (IECs), which are crucial for maintaining barrier integrity and preventing pathogen entry. Certain probiotics such as *Lactobacillus reuteri* and *Lactobacillus rhamnosus* enhance the expression and localization of TJ proteins such as ZO1, occludin, and claudin, thereby improving intestinal permeability and barrier function. They also regulate immune responses by promoting the maturation of dendritic cells, enhancing IgA secretion, and modulating inflammatory pathways. Additionally, probiotics can alter the gut microbiome composition, increase beneficial bacteria, and produce metabolites like SCFAs that further support barrier function and immune regulation [95]. The evidence for effects on intestinal permeability comes mainly from pre-clinical models. *Lacticaseibacillus rhamnosus* GG (LGG) normalizes intestinal permeability in rats with cow’s milk-induced increase in permeability [96], and it also normalizes the increased permeability caused by acute alcohol exposure [97]. This effect may be mediated in part by LGG’s propensity to adhere to the intestinal mucus layer with the adhesive protein LGG-0186 and its pili [98]. A systematic review and meta-analysis of nine studies found that probiotics and synbiotics significantly reduces serum zonulin levels, indicating improved intestinal permeability [99]. A summary of clinical trials of probiotics that have evaluated their effects on intestinal permeability is presented in Table 6 [100,101,102,103,104,105,106,107,108,109,110,111,112,113,114,115,116,117,118,119,120,121,122,123,124,125,126,127,128,129,130,131,132].

In summary, the evidence for probiotics in reducing intestinal permeability is mixed. Heterogeneity deriving from differences in study protocols, target population or disease state, probiotic formulation (as well as dosing and duration), and assessment of intestinal permeability makes it challenging to draw definitive conclusions regarding probiotics and permeability. Further large-scale, well-designed trials are needed to clarify the role of different probiotic formulations, optimal doses, and intervention durations in improving intestinal permeability across specific populations.

#### 4.2.3. Synbiotics

Descriptions and Mechanisms of Action: Synbiotics are defined as “mixture[s] comprising live microorganisms and substrate(s) selectively utilized by host microorganisms that confers a health benefit on the host” [133]. Synbiotics can be categorized into two types: complementary and synergistic. Complementary synbiotics involve a mixture where the probiotic and prebiotic components work independently to provide health benefits, whereas synergistic synbiotics involve a specific substrate that enhances the effects of the co-administered live microorganism. To be considered a synbiotic, the formulation must show evidence of health benefits when compared against a placebo and demonstrate either selective utilization by the endogenous microbiota or the administered microorganism [133].

Synbiotics support the gut epithelial barrier and intestinal permeability through multiple mechanisms, derived from their combined pre- and probiotic constituents. They enhance microbial balance by providing beneficial microorganisms (probiotics) and substrates (prebiotics), which promote the growth of beneficial bacteria and protection against pathogens. This balance leads to the production SCFAs like butyrate, which serve as an energy source for epithelial cells, enhance mucin production, and maintain tight junctions, thereby reducing intestinal permeability. Synbiotics may also regulate tight junction proteins, support mucosal immunity, reduce inflammation, and competitively exclude pathogens. These combined effects help protect and strengthen the gut barrier, ensuring its integrity and proper function [134]. A summary of clinical trials of synbiotics where effects on intestinal permeability were assessed is displayed in Table 7 [90,135,136,137,138,139,140,141,142,143,144,145,146,147,148].

The available evidence on synbiotics to reduce intestinal permeability shows varied results depending on the population and condition studied. Several studies have reported improvements in intestinal permeability with synbiotic interventions, while others have found no significant effects. Once again, interpretation is limited by considerable heterogeneity among study methodologies, target population or disease state, intervention (including dosing and duration), and assessment of intestinal permeability. Further well-designed, large-scale trials with standardized methods are needed to better understand the efficacy of synbiotics in enhancing intestinal permeability and to establish clearer guidelines for the use of specific formulations in specific conditions.

## 5. Recommendations and Conclusions

Intestinal permeability is an important component of the barrier between intraluminal antigens and the establishment of diseases of the gastrointestinal tract as well as other organs. It is significantly modified by diet, including fat and emulsifiers that increase permeability, and nutrients such as fiber, glutamine, zinc, vitamin D, polyphenols, and anthocyanins that decrease permeability. There is significant interaction of the microbiome and barrier function, including the inflammatory effects of luminal/bacterial antigens and the anti-inflammatory effects of commensals or probiotics and their products including short chain fatty acids. Epigenomic modifications of barrier functions are best illustrated by effects on junction proteins or inflammation. Further understanding of the barrier function and its potential amelioration by dietary, microbial, or, in the future, pharmacological agents will have the potential to reverse the pathobiology underpinning gastrointestinal diseases, as well as the multitude of other systemic diseases that are attributed to the leaky gut phenomenon. It is essential to use accurate measurements of intestinal permeability, particularly in vivo, and to ascertain the longitudinal changes in the barrier that occur with perturbations, disease, and amelioration through treatment.

## Figures and Tables

**Figure 1:**
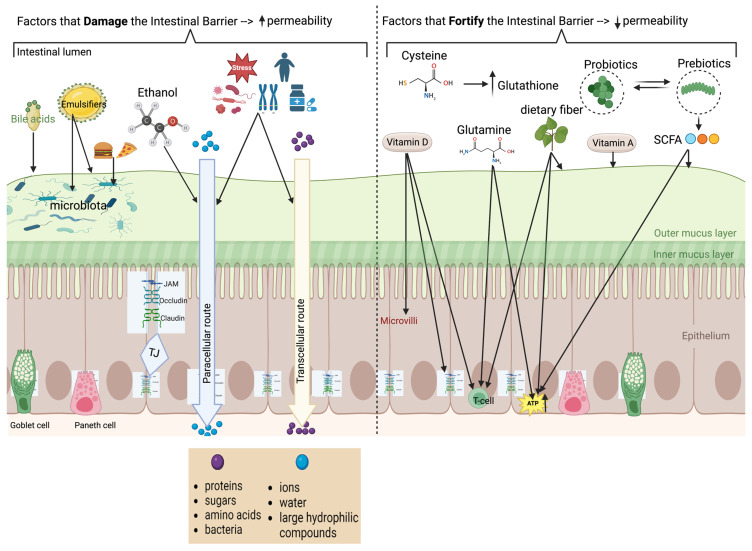
Composition of barrier, physiology of permeability, and factors which help or harm permeability (created in BioRender. matar, a. (2024) BioRender.com/m56y070 (accessed on 10 October 2024)). The left side of the figure summarizes the factors that damage the intestinal barrier resulting in increased permeability; the right side summarizes factors that enhance the intestinal barrier resulting in reduced intestinal permeability. Several amino acids, exemplified by glutamine in the figure, are associated with fortification of the intestinal barrier (as detailed in the text).

**Table 1:** Effects of high-fat diet on intestinal barrier function in humans in vivo or in vitro (adapted and updated from Camilleri and Vella [2]).

Participants	Treatment/Duration/Method	Main Results	Ref.
1015 healthy people randomly recruited; in vivo	Three-day food record; plasma LPS tested in 201 male participants.	Association between food intake (positively correlated with fat intake) and plasma LPS.	Amar et al., 2008 [43]
20 healthy adults (mean age 25 ± 3.2 y); in vivo	Control diet (olive oil, 20%) HFD with omega-3 (fish oil, 35%) HFD with omega-6 (grape seed oil, 35%); diet rich in saturated fat (coconut oil, 35%); serum endotoxin concentration	Serum endotoxin concentration increased during postprandial period after consumption of a high-saturated-fat meal, but decreased after the meal with omega-3.	Lyte et al., 2016 [44]
13 normal weight, sedentary adult males (mean age 22.2 ± 1.6 y); all weight-stable; in vivo	Two-week control diet [55% CHO, 30% fat (9% saturated), and 15% protein]; HFD for 5 days [55% fat (25% saturated fat), 30% CHO, and 15% protein]; HFM challenge [820 kcal: 25% CHO, 12% protein, 63% fat (~26% saturated fat)] pre- and post-5-day HFD; urine excretion of probe molecules.	No significant changes in gastroduodenal, small intestinal (*p* = 0.084 LM ratio), or colonic permeability following HFD.	Bowser et al., 2020 [45]
M-cell monolayers, human Peyer’s patches	Soluble NSP and food emulsifiers. Polysorbate-80 at 0.01% and 0.1%vol/vol *E. coli* translocation.	Polysorbate-80 increased *E. coli* translocation across M cells and human Peyer’s patches.	Roberts et al., 2010 [46]

CHO: carbohydrate; LPS: lipopolysaccaride, HFD: high-fat diet, HFM: high-fat meal, LM ratio: lactulose–mannitol ratio; NSP: non-starch polysaccaride.

**Table 2:** Summary of preclinical models of altered permeability investigated in vivo (adapted and updated from Khoshbin and Camilleri [42]).

Component	Preclinical Models	Effects in Preclinical Models	Reference
Dietary Component			
Dietary Fiber	- Resistant oligosaccharides: Mistry et al. (male mice on Western-type diet, in vivo); - Non-starch polysaccharides: in vivo study with inulin (healthy male volunteers); - Resistant starches: Gondalia et al., in vivo study (healthy adults with high- vs. low-amylose wheat).	Supplementation reduced bodyweight gain, fat accumulation, insulin resistance, altered gut microbiota, lowered lactulose/mannitol ratio and serum zonulin, increased mucosal GLP-2; Similar effects on fecal output and SCFA excretion; HAW-R increased fecal butyrate.	Mistry et al., 2020 [47] Camilleri et al., 2022 [21]
Vitamin D	- VDR-deficient mice, in vivo:	Increased susceptibility to bacteria and LPS, weakened tight junctions.	Yamamoto et al., 2020 [49]
Minerals: Zinc	- Zinc deficiency: Omry et al., in vivo study (chicks); - Zinc deficiency [51]; - Zinc overdose: chronic exposure in mice, in vivo study.	Mixed evidence on potential to upregulate “leaky” junctions in some contexts; Reduced microbial diversity, impaired nutrient uptake.	Yamamoto et al., 2020 [49] Koren et al.,2020 [51]
Others: polyphenols, flavanones, anthocyanins	- Urolithins: Administration of Uro-A and Uro-B in rats (in vivo study) on a high-fat diet.	Altered gut microbiota, decreased microbes associated with weight and inflammation.	Abdulrahman et al., 2021 [53]
Arabinoxylans	Overweight and obesity	Effects on gut health detailed in the body of the text.	
Probiotics	Obesity; primary sclerosing cholangitis and IBD.		
Synbiotics	Indomethacin-induced increased permeability; obesity.		
Prebiotics	Non-obese diabetic mice; Western diet induced increased intestinal permeability in mice; aspirin-induced intestinal permeability in humans; children with type 1 diabetes mellitus; pre-term infants; burns.		
Macronutrients			
Protein	Protein-restricted diet or dexamethasone-induced permeability in chickens; Dietary meat and fish (and fat) in epidemiological studies of IBD.	Increased permeability and bacterial translocation; Increased risk of IBD.	Khoshbin et al., 2020 [42]
Sulfur-containing AAs: cysteine & methionine	Rats infected with *Salmonella enteritidis*; ischemia-reperfusion injury in rats; high-fat diet with methionine restriction in mice.	Effects on gut health detailed in Figure 1.	
Other AAs: Glutamine, L-tryptophan, Arginine	- Glycyl-glutamine (GlyGln): in vivo study with weaned piglets; - Ala-Gln and glutamine (Gln): in vivo study in mice with DSS-induced colitis; - Tryptophan: colitis in mice. - Arginine: intestinal injury induced by heat stress; ischemia reperfusion; methotrexate; NASH or intestinal obstruction in rodents; mouse colitis.	Improved ileum morphology, reduced inflammation, enhanced oxidative status, Both improved colitis symptoms, with Ala-Gln showing superior efficacy; Evidence suggests benefits in gut health and barrier function.	Xu et al., 2021 [54] Xu et al., 2021 [55]
Fat:	Fat-restricted diets.	Alterations in microbiota and further explanations in Table 1.	
Alcohol	Alcohol abuse and alcoholic liver disease.	Increased permeability and bacterial translocation.	Khoshbin et al., 2020 [42]
Intraluminal Emulsifiers			
Endogenous bile acid	Mammalian colon	Detergent effects with increased permeability measured by PEG 400 and scanning EM.	Khoshbin et al., 2020 [42]
Dietary emulsifiers	Mouse colon permeability and inflammatory changes.	Effects on gut health detailed in the text.	
Receptors Associated with Intraluminal Factors			
Aryl hydrocarbon receptor	Diverse animal models of immune diseases.	Anti-inflammatory	Khoshbin et al., 2020 [42]

AA: amino acids; DSS: dioctyl sodium sulfosuccinate; EM electron microscopy; IBD: inflammatory bowel disease; IBS-D: diarrhea-predominant irritable bowel syndrome; Ala-Gln: alanyl glutamine; DSS: dextran sodium sulfate; GLP-2: glucagon-like peptide 2; Gln: glutamine; GlyGln: glycyl-glutamine; HAW R: high-amylose wheat resistant starch; NAFLD: non-alcoholic fatty liver disease; NASH: non-alcoholic steatohepatitis; Uro-A: urolithin A; Uro-B: urolithin B; VDR: vitamin D receptor.

**Table 3:** Summary of preclinical models investigating human disease states of altered permeability (adapted and updated from Khoshbin and Camilleri [42]).

Component	Human Diseases	Effects in Human Diseases	Reference
Dietary Component			
Dietary Fiber	Effect on metabolic markers and gut health detailed in text.		Gondalia et al., 2022 [48]
Vitamin D	In vivo study with high levels of active vitamin D.	Associated with gut bacteria producing butyrate, enhancing intestinal barrier; Mixed evidence on potential to upregulate “leaky” junctions in some contexts.	Thomas et al., 2020 [50] Yamamoto et al., 2020 [49]
Minerals: Zinc	Zinc deficiency linked to gastrointestinal disorders; appropriate dosing improves gut bacteria biodiversity and barrier integrity.	Increased oxidative stress, compromised barrier integrity.	Skalny et al., 2021 [52]
Others: polyphenols, flavanones, anthocyanins	Limited direct studies on permeability.	Polyphenols show general gut health benefits (see text).	
Macronutrients			
Protein	Dietary meat and fish (and fat) in epidemiological studies of IBD.	Increased risk of IBD	Khoshbin et al., 2020 [42]
Other AAs: Glutamine, L-tryptophan, Arginine	Limited direct studies.	Evidence suggests benefits in gut health and barrier function	Xu et al., 2021 [55]
Fat:	Fat-restricted diets.	Alterations in microbiota and further explanations in Table 1	Xu et al., 2021 [54]
Alcohol	Alcohol abuse and alcoholic liver disease.	Increased permeability and bacterial translocation	Khoshbin et al., 2020 [42]

AA: amino acids; IBD: inflammatory bowel disease.

**Table 4:** Summary of nutrients increasing or decreasing intestinal permeability and proposed mechanisms.

Nutrient	Proposed Mechanisms	Factors That Decrease Permeability	Factors That Increase Permeability	References
Dietary fiber	- Fermentation by gut microbiota produces SCFAs (e.g., acetate, butyrate, propionate); - SCFAs enhance tight junction integrity and modulate inflammation.	- SCFAs increase production of mucins and enhance epithelial cell function; - Butyrate supports mucosal health and decreases inflammation.	- Inadequate fiber intake can lead to reduced SCFA production and compromised barrier function.	[21,47,48,56,57,58,59,60,61,62]
Polyphenols	- Antioxidant properties; - Modulation of gut microbiota; - Reduction in oxidative stress and inflammation.	- Urolithin A and B reduce inflammation and oxidative stress, improving barrier function; - Anthocyanins provide antioxidant protection.	- Variable metabolism of polyphenols leading to inconsistent effects across individuals.	[53,63,64,65,66,67]
Glutamine	- Supports synthesis of tight junction proteins; - Enhances gut microbiota diversity and function; - Reduces inflammation.	- GlyGln and Gln supplementation improves intestinal morphology and barrier integrity; - Support for mucosal health and reduction in inflammation.	- High doses of supplementation could disrupt balance and exacerbate inflammation.	[54,55]
Vitamin D	- Enhances expression of tight junction proteins (e.g., ZO-1, occludin, claudin-1); - Upregulates antimicrobial peptides.	- Adequate levels of active vitamin D strengthen the epithelial barrier; - Supports microbiota associated with butyrate production.	- Potential to upregulate “leaky” junctions in some cases; - Over-supplementation may disrupt barrier function.	[49,50,68]
Zinc	- Influences tight junction protein expression; - Modulates gut microbiota diversity; - Affects immune responses.	- Optimal zinc levels improve barrier integrity and gut microbiota diversity; - Enhances nutrient uptake and reduces permeability.	- Zinc deficiency leads to dysbiosis and compromised barrier function; - Overdosing induces oxidative stress and disrupts barrier integrity.	[51,52,69,70]
SCFAs	- Produced from fermentation of dietary fiber by gut microbiota; - SCFAs (acetate, butyrate, propionate) enhance tight junction integrity and reduce inflammation.	- Butyrate is particularly beneficial in reducing inflammation, supporting mucosal health, and increasing tight junction protein expression; - SCFAs lower colonic pH, inhibiting pathogenic bacteria.	- Reduced SCFA production due to low fiber intake can compromise barrier integrity and gut health.	[58,59,60,61,62]

SCFAs: short-chain fatty acids, GLN: glutamine; GLY: glycine; ZO-1: zonula occludens.

**Table 5:** Study characteristics and findings of clinical trials of prebiotics where effects on intestinal permeability were among the outcomes assessed.

Sample	Treatment/Duration	Main Results	Ref.
Formula-fed infants and exclusively breast-fed infants	Formula supplementation with a prebiotic mixture of galacto-oligosaccharides and inulin for 10 weeks	Reduced IP (assessed using the lactulose-to-mannitol ratio) and improved microbiota profiles	Francavilla et al., 2006 [80]
Preterm infants	Enteral supplementation with a prebiotic mixture (1.5 g per kg per day of small-chain galacto-oligosaccharides, long-chain fructo-oligosaccharides, and nonhuman milk acidic oligosaccharides) for 30 days	No effect on IP (L:M ratio)	Westerbeek et al., 2010 [81]
Children with type 1 diabetes	8 g of oligofructose-enriched inulin (chicory root) for three months	Reduced IP (L:M ratio), increased C-peptide levels, induced favorable changes in gut microbiota composition	Ho et al., 2019 [82]
Adult burn patients	6 g of oligofructose for 15 days	No effect on IP (L:M ratio and sucrose excretion)	Olguin et al., 2005 [83]
Healthy young men	Inulin-enriched pasta for 5 weeks	Reduced IP (L:M ratio) and decreased serum levels of zonulin and GLP-2	Russo et al., 2012 [84]
Adults with overweight or obesity	Arabinoxylan (7.5 or 15 g per day) for 6 weeks	No effect on IP (multi-sugar test), but upregulation of gene transcription of tight junction proteins, decreased microbiome diversity (though without compositional change), decreased fecal pH, increased fecal concentrations of total SCFAs, acetate, propionate and butyrate, decreased TNFα production	Salden et al., 2018 [85]
Adults with slow gastrointestinal transit without constipation	15 g of wheat bran extract arabinoxylan–oligosaccharide (AXOS) for 12 weeks	No effect on IP (multi-sugar test), improved stool consistency, increased *Bifidobacterium* levels, no effect on whole-gut transit time or energy metabolism	Müller et al., 2020 [86]
Elderly adults	12 g per day oat β-glucan or wheat arabinoxylan for 6 weeks	No effect on indomethacin-induced increase in IP (multi-sugar test) or microbiota composition	Ganda Mall et al., 2020 [87]
Adults with overweight or obesity	25 g of yacon flour (rich in phenolic compounds and fructooligosaccharides) plus an energy-restricted diet for 6 weeks	No effect on IP (multi-sugar test), increased plasma antioxidant capacity, deceased oxidative stress markers, no effect on fecal short-chain fatty acids or inflammation markers	Machado et al., 2020 [88]
Adult men with type II diabetes	5.5 g per day of galacto-oligosaccharides for 12 weeks	No effect on IP (by urinary recovery of oral 51Cr-EDTA), glucose tolerance, or microbial community structure	Pedersen et al., 2016 [89]
Adults at risk for type II diabetes	10 g per day of inulin for 6 weeks	No effect on IP (multi-sugar test), reduced fasting insulin levels, improved HOMA-IR, increased fecal Bifidobacteria, no effect on plasma endotoxin concentrations or lipopolysaccharide-binding protein concentrations	Mitchell et al., 2021 [90]
Adults with metabolic syndrome	Dietary Guidelines for Americans (DGA)-based diet incorporating potatoes (17.5 g per day of resistant starch) for 2 weeks	Reduced small IP (L:M ratio) and postprandial endotoxemia, no changes in cardiometabolic markers	Cao et al., 2022 [91]
Women with obesity and major depressive disorder	10 g per day of inulin	No effect on IP, depressive symptoms, inflammatory biomarkers, or weight	Vaghef-Mehrabani et al., 2023 [92]
Systematic review	Chicory inulin	Reduced IP	Nascimento et al., 2024 [3]

IP = intestinal permeability.

**Table 6:** Study characteristics and findings of clinical trials of probiotics where effect on intestinal permeability was among the outcomes assessed. Specific information about the microbiota in diverse, commercial formulations is included in the Supplementary Material.

Sample	Treatment/Duration	Main Results	Ref.
Preterm Infants	Fortification of formula with *Bifidobacter lactis* (2 × 10^7^ cfu/g of dry milk) for 30 days	Reduced IP (L:M ratio) and increased head growth	Stratiki et al., 2007 [100]
Children with rotavirus or cryptosporidial diarrhea	Culturelle *Lacticaseibacillus rhamnosus* GG (ATCC 53103) (1 × 10^10^ CFU) for 4 weeks	Reduced IP (L:M ratio) and improved clinical outcomes	Sindhu et al., 2014 [101]
Children with Crohn disease	Valio LGG (1 × 10^10^ CFU twice daily) for 6 months	Reduced IP (double sugar test) and improved Crohn disease severity scores	Gupta et al., 2000 [102]
Children with atopic dermatitis	*Lactobacillus rhamnosus* 19070-2 (1 × 10^10^ CFU) and *L reuteri* DSM 12246 (1 × 10^10^ CFU) for 6 weeks	Reduced IP (L:M ratio), improved gastrointestinal symptoms; association between improved permeability and reduced eczema.	Rosenfeldt et al., 2004 [103]
Children with short bowel syndrome	Culturelle LGG (1 × 10^9^ CFU) for 4 weeks	No effect on IP (L:M ratio), fecal *Lactobacillus* colonization, or small intestinal bacterial overgrowth	Sentongo et al., 2008 [104]
Healthy adults	Dairy product containing live or heat-inactivated *Lactobacillus GG* (>10^7^/mL), *Lactobacillus helveticus* (>10^7^/mL), and *Lactobacillus acidophilus* (>10^7^/mL) (2.4 × 10^9^ CFU) for 10 days	Live but not heat-killed probiotics reduced indomethacin-induced alterations in GP (urinary excretion of sucrose and L:M ratio) but did not affect IP	Gotteland, Cruchet, and Verbeke 2008 [105]
Healthy adults	De Simone formulation probiotic (900 billion bacteria per day) for 21 days	Reduced fecal calprotectin concentrations during indomethacin therapy	Montalto et al., 2010 [106]
Healthy adults	Yogurt enriched with *Lactobacillus gasseri* OLL2716 (1 × 10^9^ CFU) for 16 weeks	Reduced IP (urinary sucrose excretion) and the likelihood of a positive fecal occult blood test after both high and low doses of aspirin	Akama et al., 2011 [107]
Healthy adults	Lallemand *Lacticaseibacillus rhamnosus* R0011 and *Lactobacillus helveticus* R0052 (at least 4 billion CFU)	Did not prevent the increase in gastroduodenal permeability (urinary sucrose excretion) induced by an acute aspirin challenge	Judkins et al., 2024 [108]
Adults with Crohn disease in remission	Floratil *Saccharomyces boulardii* (4 × 10^8^ CFU 3 times per day) for three months	Reduced IP (L:M ratio), although did not normalize it	Garcia Vilela et al., 2008 [109]
Adults with ulcerative colitis in remission	Ecologic 825 formulation (1.5 × 10^10^ CFU) for 12 weeks	No effect on IP (multi-sugar test), serum zonulin, fecal zonulin, inflammation markers, or stool characteristics	Wegh et al., 2019 [110]
Adults with diarrhea-predominant IBS	Probiotic fermented milk for 4 weeks	Reduced small IP (L:M ratio) and reduced IBS symptoms, though did not affect colonic permeability (sucralose excretion)	Zeng et al., 2008 [111]
Adults with IBS	Zircombi *Bifidobacterium longum* BB536 (4 billion CFU), *Lactobacillus rhamnosus* HN001 (1 billion CFU), and vitamin B6 for 30 days	Reduced IP (sucralose excretion), improved IBS symptoms, and favorably modified the gut microbiota and metabolome	Bonfrate et al., 2020 [112]
Adults with IBS	De Simone formulation (900 billion CFU) for 4 or 8 weeks	Reduced IP (multi-sugar test) and improved IBS symptoms	Boonma et al., 2021 [113]
Adults with IBS-D and confirmed increased permeability	Lactibiane Tolérance formulation (1 × 10^10^ CFU twice per day) for 30 days	Reduced (81.5%) or normalized (37%) IP (radionucleotide tracers) and improved IBS symptoms, including abdominal pain and stool consistency, while also enhancing QOL	Ait Abdellah et al., 2023 [114]
Adults with IBS-D	Lactibiane Tolérance formulation (10 × 10^9^ CFU twice daily) for 4 weeks	While IP (urinary lactulose excretion) remained unchanged, the probiotic improved IBS symptoms, QOL, and gut microbiota. In responders with reduced diarrhea, transcellular permeability significantly decreased, and paracellular permeability trended toward a decrease, though overall permeability showed no significant change.	Marchix et al., 2023 [115]
Meta-analysis of patients with small intestinal bacterial overgrowth	Various	No effect on intestinal permeability	Zhong et al., 2017 [116]
Adults with metabolic syndrome	Yakult (milk beverage fortified with 10^8^ CFU/mL *Lactobacillus casei* Shirota) three bottles per day for 3 months	No effect on intestinal permeability (sugar absorption test and diaminooxidase serum levels), endotoxin levels, or neutrophil function	Leber et al., 2012 [117]
Systematic review of 12 animal and 14 clinical studies in obesity	Various, especially with *Bifidobacterium*, *Lactobacillus*, and *Akkermansia*	Often reduces IP in obesity, although clinical results are inconsistent	DiMattia et al., 2014 [118]
Adult patients with cirrhosis and a hepatic venous pressure gradient > 10	De Simone formulation (3600 billion bacteria daily) for 2 months	No effect on IP (L:M ratio, sucrose and sucralose tests) or portal pressure	Tandon et al., 2009 [119]
		No effect on IP (endotoxin levels), cytokine levels, microbiota or HVPG, but decrease in aldosterone	Jayakumar et al., 2013 [120]
Adults with chronic liver disease	Duolac Gold formulation (5 × 10^9^ CFU twice daily) for 4 weeks	No effect on IP (L:M ratio), alleviated SIBO, improved GI symptoms	Kwak et al., 2014 [121]
Adults with cirrhosis primary and secondary analysis	Ecologic Barrier formulation (1.5 × 10^10^ CFU per day) for 6 months	No effect on IP (L:M ratio) or bacterial translocation, but did improve some immune and liver function, seen by increased serum neopterin levels and enhanced neutrophil reactive oxygen species production	Horvath et al., 2016 [122]
		Reduced IP (increased neopterin and decreased fecal zonulin), enriched the gut microbiota, and increased levels of beneficial bacteria	Horvath et al., 2020 [123]
Adults with metabolic dysfunction-associated steatotic liver disease	HEXBIO Microbial Cell Preparation (30 billion CFU twice daily) for 6 months	No effect on IP (zonulin and zonula occluden-1 (ZO-1) levels) or gut microbial diversity, although did induce changes in microbiota composition and decreases in IFN-γ and TNF-α	Ayob et al., 2023 [124]
Adults with acute pancreatitis	Enteral feeding supplemented with *Lactobacillus plantarum* (1 × 10^8^ CFU) for 7 days	Reduced IP (L:M ratio) and improved clinical outcomes including reduced colonization by pathogenic organisms and fewer septic complications	Qin et al., 2008 [125]
Adults with severe acute pancreatitis	Ecologic 641 formulation (1 × 10^10^ CFU twice daily) for 28 days	No effect on IP (PEG permeability test), increased enterocyte damage and bacterial translocation among those with organ failure (a subsequent study showed increased mortality with this probiotic in acute pancreatitis) [122]	Besselink et al., 2009 [126]
Critically ill adults	Proviva oatmeal and fruit drink containing 5 × 10^7^ CFU/mL of *Lactobacillus plantarum* 299v 500 mL per day until hospital discharge or patient preference to discontinue	No effect on IP L:M ratio), gastric microbiota composition, endotoxin levels, sepsis morbidity or mortality, but reduced serum IL-6 levels	McNaught et al., 2005 [127]
Critically ill adults	De Simone formulation 900 billion CFU twice daily or inactivated probiotic sonicates	No effect on IP (L:M ratio), but increased systemic IgA and IgG in live but not dead bacteria group	Alberda et al., 2007 [128]
Adults with HIV on antiretroviral therapy	De Simone formulation 900 billion CFU once daily for 4 weeks followed by twice daily to 26 weeks	No effect on IP (assessed by colonic biopsies measuring CD4+ cells, IL-17+ cells, and myeloperoxidase), systemic inflammation, or cellular markers of inflammation, although it did alter the microbiome by increasing beneficial species and reducing *Gammaproteobacteria*	Presti et al., 2021 [129]
Pregnant women with overweight	*Bifidobacterium animalis* ssp. *lactis* 420 (1 × 10^10^ CFU) and *Lactobacillus rhamnosus* HN00 (1 × 10^10^ CFU) and/or LC-PUFA for 21 weeks (on average)	No protective effect on the increase in IP during pregnancy (serum zonulin and LPS)	Mokkala et al., 2018 [130]
Elderly adults	*Lactobacillus paracasei* HII01 (2 × 10^10^ CFU), *Bifidobacterium breve* (2 × 10^10^ CFU), and *Bifidobacterium longum* (1 × 10^10^ CFU)	Reduced IP (assessed using a colorimetric urine analysis), enhanced HDL-cholesterol levels, and positively affected obesity-related biomarkers and short-chain fatty acids	Chaiyasut et al., 2022 [131]
Adults with migraine	Ecologic barrier formulation 5 × 10^9^ CFU daily for 12 weeks	No effect on IP (L:M ratio and fecal and serum zonulin levels) or migraine severity or frequency	de Roos et al., 2017 [132]

IP = intestinal permeability; GP = gastric permeability GI = gastrointestinal QOL = quality of life.

**Table 7:** Study characteristics and findings of clinical trials of synbiotics where effects on intestinal permeability were among the outcomes assessed. Specific information about the microbiota in diverse, commercial formulations is included in the Supplementary Material.

Sample	Treatment/Duration	Main Results	Ref.
Critically ill adults	Trevis probiotic (4 × 10^9^ of *Lactobacillus acidophilus* La5, *Bifidobacterium lactis* Bb 12, *Streptococcus thermophilus*, *Lactobacillus bulgaricus*) plus 7.5 g of oligofructose twice daily for 8 days	No effect on IP (L:M ratio), septic complications, or mortality, but reduced the incidence of potentially pathogenic bacteria in nasogastric aspirates.	Jain et al., 2004 [90]
Adults undergoing elective colorectal surgery	Mechanical bowel preparation, neomycin, and Trevis probiotic three times per day plus 15 g oligofructose twice daily	No effect on small IP (L:M ratio), whole gut permeability (sucralose excretion), inflammatory response, or sepsis morbidity, but there was significantly reduced fecal *Enterobacteriaceae* and bacterial translocation.	Reddy et al., 2007 [135]
Adult critically ill trauma patients	Synbiotic 2000 (4 × 10^10^ CFU) plus 2.5 g of each of β glucan, inulin, pectin, and resistant starch for 7 days	Reduced IP (L:M ratio) and incidence of infections, including pneumonia.	Spindler-Vesel et al., 2007 [136]
Adults with acute pancreatitis	2.5 billion CFU of *Lactobacillus acidophilus*, *Bifidobacterium longus*, *Bifidobacterium bifidum*, and *Bifidobacterium infantalis* with 25 mg of fructo-oligosaccharide for 7 days	No effect on IP (L:M ratio), endotoxemia, duration of hospital or intensive care unit stay, or mortality, but decreased levels of C-reactive protein and immunoglobulins.	Sharma et al., 2011 [137]
Healthy adults	Biosource Gut Balance (2 × 10^9^ CFU) plus 200 mg immunoglobulin, 50 mg lactoferrin, 90 mg raftiline, and 10 mg raftilose ) for 21 days	No effect on IP (uncertain how this was assessed apart from some measure via fecal and urine samples), fecal SCFAs, or markers of mucosal immunity.	West et al., 2012 [138]
Adults with esophageal cancer undergoing esophagectomy	Yakult 400 (at least 4 × 10^10^ *Lactobacillus casei* Shirota) Yakult Honsha (at least 1 × 10^10^ *Bifidobacterium breve* Yakult), and 15 g galacto-oligosaccharides for 7 days following surgery	Reduced IP (indirectly assessed via reduced bacterial translocation to mesenteric lymph nodes and reduced incidence of postoperative bacteremia).	Yokoyama et al., 2014 [139]
Healthy adults	Ecologic 825 (6 g per day) combined with 10 g per day fructooligosaccharides for 2 weeks	No effect on IP (multi-sugar test) at baseline or after indomethacin, though stool frequency increased.	Wilms et al., 2016 [140]
Adults with overweight	*Bifidobacterium animalis* ssp. *lactis* 420 B420 1 × 10^10^ CFU per day combined with 12 g of ultra polydextrose for 6 months	Reduced effect on IP (plasma zonulin levels; reduction in zonulin correlated with reductions in trunk fat mass), reduced body fat mass, waist circumference, and energy intake, increased fecal SCFAs.	Stenman et al., 2016 [141]
Adults with MASLD	Nestle Fiber Mais Flora *Lactobacillus reuteri* 1 × 10^8^ CFU plus 4 g of guar gum and inulin twice daily combined with nutritional counseling for three months	No effect on IP (L:M ratio) or LPS levels, but reduced hepatic steatosis, weight, BMI, and waist circumference.	Ferolla et al., 2016 [142]
Adults with IBS-D	OMNi-BiOTiC Stress Repair (6 × 10^10^ CFU) plus corn starch, maltodextrin, inulin, fructooligosaccharides, and amylases) for 4 weeks	Reduced IP (fecal zonulin levels), improved IBS symptoms, increased microbial diversity in the upper GI tract, reduced CD4+ T cells in the ascending colon, and elevated fecal SCFAs.	Moser et al., 2018 [143]
Adults on long-term proton pump inhibitor therapy	OMNi-BiOTiC PPI (24 × 10^9^ CFU) in a prebiotic matrix (composed of corn starch, maltodextrin, fructo-oligosaccharide P6, inulin P2, and vegetable protein) for 3 months	Reduced IP (reflected by a reduction in fecal zonulin) in those with increased permeability at baseline, which was maintained three months post-intervention; no significant changes were observed in other markers of IP, including lipopolysaccharide, lipopolysaccharide-binding protein, and sCD14 levels.	Horvath et al., 2020 [144]
Adults with obesity and type II diabetes	Ecologic barrier formulation with fructo-oligosaccharides P6, and konjac glucomannan P13 for 6 months	Reduced IP (serum zonulin), lipoprotein (a), and hip circumference, with no changes in glucose metabolism.	Horvath et al., 2020 [145]
Adults with stage III-IV chronic kidney disease	NATUREN G (4.8 × 10^9^ CFU), 2.5 g fructoligosaccharides, 2.5 g inulin, and antioxidants quercetin, resveratrol, and proanthocyanidins for 2 months	Reduced small IP (assessed using urinary recovery of oligosaccharide probes after oral administration), alleviated GI symptoms, and reduced free serum indoxyl sulfate..	Cosola et al., 2021 [146]
Adult women with migraine	Familact (1 × 10^9^ CFU) plus 21 mg fructooligosaccharides for 12 weeks	Reduced IP (serum zonulin), frequency of migraine attacks, painkiller use, GI symptoms, and CRP.	Ghavami et al., 2021 [147]
Adults with coronary artery disease	Inulin 15 mg or LGG 1.9 × 10^9^ CFU or their combination for 60 days	Reduced IP (LPS), IL-6, and TLR-4 levels while increasing serum total antioxidant capacity.	Liu et al., 2024 [148]

IP = intestinal permeability; GI = gastrointestinal.

## Data Availability

This narrative review is based on the reported literature search, with no original data presented.

## Supplementary Materials

The following supporting information can be downloaded at: https://www.mdpi.com/article/10.3390/nu16203494/s1, File S1.
