# Supplementary material for: Intestinal Barrier Impairment, Preservation, and Repair: An Update
Source: Nutrients. 2024 Oct 15;16(20):3494. doi: 10.3390/nu16203494 (PMC11509958; doi:10.3390/nu16203494)
Supplement: Supplementary file 1 [file nutrients-16-03494-s001.zip › nutrients-3242609-supplementary.pdf]

<u>Supplemental Material for Table 6 in the article</u>

De Simone formulation probiotic (total 900 billion bacteria):
Lactobacillus paracasei DSM24733/SD5218
Lactobacillus plantarum DSM 24730/SD5209
Lactobacillus acidophilus DSM 24735/ SD5212
Lactobacillusdelbrueckii subspecies *bulgaricus* DSM 24734/SD5210
Bifidobacterium longum DSM 24736/SD5219
Bifidobacterium infantis DSM 24737/SD5220
Bifidobacterium breve DSM 24732/SD5206
Streptococcus thermophilus DSM 24731/SD5207
total 900 billion bacteria

Ecologic 641 formulation
Lactobacillus plantarum WCFS1
Lacticaseibacillus rhamnosus GR1
Lactobacillus acidophilus La-14
Bifidobacterium bifidum Bb-02
Bifidobacterium lactis BL-04
Bifidobacterium longum BB536)
total 1 x 10^{10} CFU

Ecologic 825 formulation:
Bifidobacterium bifidum W23
Bifidobacterium lactis W51
Bifidobacterium lactis W52
Lactobacillus acidophilus W22
Lactobacillus casei W56
Lactobacillus paracasei W20
Lactobacillus plantarum W62
Lactobacillus salivarius W24
Lactococcus lactis W19
total 1.5 $*$ 10^{10} CFU

Ecologic Barrier formulation
Bifidobacterium bifidum W23
Bifidobacterium lactis W52
Lactobacillus acidophilus W37
Lactobacillus brevis W63
Lactobacillus casei W56
Lactobacillus salivarius W24
Lactococcus lactis W19
Lactococcus lactis W58
total 1.5 x 10^{10} CFU

Lactibiane Tolérance formulation
B. lactis LA 303
B. lactis LA 304
L. acidophilus LA 201
L. plantarum LA 301
L. salivarius LA 302
total 1 x 10^{10} CFU

Duolac Gold formulation
Bifidobacterium bifidum (KCTC 12199BP)
Bifidobacterium lactis (KCTC 11904BP)
Bifidobacterium longum (KCTC 12200BP)
Lactobacillus acidophilus (KCTC 11906BP)
Lactobacillus rhamnosus (KCTC 12202BP)
Streptococcus thermophilus (KCTC 11870BP)
total 5x10^9 CFU

HEXBIO Microbial Cell Preparation
Lactobacillus acidophilus BCMC 12130
Lactobacillus casei subsp.BCMC 12313
Lactobacillus lactis BCMC 12451
Bifidobacterium bifidum BCMC 02290
Bifidobacterium infantis BCMC® 02129
Bifidobacterium longum BCMC 02120
total 30 billion CFU

Probiotic fermented milk
Streptococcus thermophilus 1 x10^8 CFU
Lactobacillus bulgaricus 1 x 10^7 CF
Lactobacillus acidophilus 1.0 x 10^7 CFU
Bifidobacterium longum 1 *x* 10^7 CFU
Total 130 million CFU/ml

<u>Supplemental Material for Table 7 in the article</u>

Synbiotic 2000
Pediococcus pentosaceus 5–33:3 1 x 10^{10} CFU
Lactococcus raffinolactis 32–77:1 1 x 10^{10} CFU
Lactobacillus paracasei subsp *paracasei* 19 1 x 10^{10} CFU
Lactobacillus plantarum 2362 1 x 10^{10} CFU
Total 4 x 10^{10} CFU

Biosource Gut Balance
Lactobacillus paracasei subs *Paracasei* 431 4.6 × 10^8 CFU
Bifidobacterium animalis ssp *lactis* BB-12 6 × 10^8 CFU
Lactobacillus acidophilus LA-5 , 4.6 × 10^8 CFU
LGG 4.6 × 10^8 CFU
Total 2 x 10^9 CFU

Ecologic 825 formulation:
Bifidobacterium bifidum W23
Bifidobacterium lactis W51
Bifidobacterium lactis W52
Lactobacillus acidophilus W22
Lactobacillus casei W56
Lactobacillus paracasei W20
Lactobacillus plantarum W62
Lactobacillus salivarius W24
Lactococcus lactis W19
total 1.5 ∗ 10^{10} CFU

OMNi-BiOTiC Stress Repair (7.5 × 10^9 CFU of each)

Lactobacillus casei W56
Lactobacillus acidophilus W22
Lactobacillus paracasei W20
Lactobacillus salivarius W24
Lactobacillus plantarum W62
Lactococcus lactis W19
*Bifidobacterium lacti*s W51 and W52
Bifidobacterium bifidum W23
Total 6 x 10^{10} CFU

OMNi-BiOTiC PPI (4 g of 2 x 10^9 CFU per g)
Bacillus coagulans W183
Bacillus subtilis W201
Bifidobacterium bifidum W23
Bifidobacterium lactis W52
Bifidobacterium lactis W5
Lactobacillus acidophilus W37
Lactobacillus acidophilus W22
Lactobacillus casei W56
Lactobacillus rhamnosus W71
Lactobacillus salivarius W24
Lactococcus lactis W19
Propionibacterium freudenreichii W200
Total 24 x 10^9 CFU

NATUREN G
Lactobacillus casei LC4P1 2.4 x 10^9 CFU
Bifidobacterium animalis BLC1 2.4 x 10^9 CFU
Total 4.8 x 10^9 CFU

Familact (1 x 10^9 of each)
Lactobacillus casei
Lactobacillus acidophilus
Lactobacillus rhamnosus
Lactobacillus helveticus
Lactobacillus bulgaricus
Lactobacillus plantarum
Lactobacillus gasseri
Bifidobacterium breve
Bifidobacterium longum
Bifidobacterium lactis
Bifidobacterium bifidum
Streptococcus thermophilus
Total 12 x 10^9 CFU
